# Factors Influencing Acceptance of Personal Health Record Apps for Workplace Health Promotion: Cross-Sectional Questionnaire Study

**DOI:** 10.2196/16723

**Published:** 2020-06-04

**Authors:** Hyun Sang Park, Kwang Il Kim, Jae Young Soh, Young Ho Hyun, Sae Kyun Jang, Sol Lee, Ga Young Hwang, Hwa Sun Kim

**Affiliations:** 1 Digital Healthcare Department BIT Computer Co Ltd Seoul Republic of Korea; 2 Department of Medical Informatics Kyungpook National University Daegu Republic of Korea; 3 Finance Programs Department Korea Occupational Safety & Health Agency Ulsan Republic of Korea; 4 Research Institute HealthConnect Co Ltd Seoul Republic of Korea; 5 Elecmarvels Co Ltd Daegu Republic of Korea

**Keywords:** personal health record app, workplace health promotion, unified theory of acceptance and use of technology, perceived risk

## Abstract

**Background:**

Health care technologies can help improve workers’ health and productivity by supporting workplace health promotion. A personal health record app is used to manage medical data such as results from medical checkups, which facilitates decision making for medical personnel. However, an analysis of users’ technology acceptance is required to provide appropriate services based on personal health record apps.

**Objective:**

The purpose of this study was to analyze the factors influencing the behavioral intention of health experts and workers to use an app in workers’ health centers and to examine differences in their perception of the main variables.

**Methods:**

The study involved health experts and workers who visited 21 workers’ health centers in Korea to verify a research model in which perceived risk was added to the unified theory of acceptance and use of technology, a representative theory of information technology acceptance. After receiving ethical approval from the Korea National Institute for Bioethics Policy, 1050 questionnaires were distributed over 7 weeks with cooperation of the Korea Occupational Safety and Health Agency. A multiple linear regression analysis and multigroup path analysis were performed to verify the hypotheses, and independent samples t tests were performed to analyze differences between workers’ and health experts’ perception of the main variables.

**Results:**

The analysis included data from 866 respondents (687 workers and 179 health experts). Effort expectancy (beta=.08, *P*=.03), social influence (beta=.43, *P*<.001), performance expectancy (beta=.07, *P*=.008), and facilitating conditions (beta=.13, *P*<.001) exerted significant positive effects on behavioral intention, whereas perceived risk (beta=–.29, *P*<.001) exerted a significant negative effect on behavioral intention. Performance expectancy had a significant effect on path differences depending on gender (critical ratio=–3.38) and age (critical ratio=1.97). Workers’ mean scores for the main variables were higher relative to those of health experts for all remaining variables except perceived risk, and significant differences were observed for all remaining variables except facilitating condition.

**Conclusions:**

Social influence exerted the strongest effect on behavioral intention to use the personal health record app. Consequently, it is necessary to coordinate health promotion activities in the workplace as well as the operational direction of community institutions such as in workers’ health centers to allow workers to manage their own health via continuous use of the app. In addition, the app should be developed based on a requirement analysis of the balance between both interest groups in consideration of differences in perspective between consumers and service providers.

## Introduction

### Background

Many workers spend most of their waking hours in the workplace [1], which is an environment that can have both positive and negative effects on health [2]. As such, the workplace is the best environment to apply the concept of health promotion. The World Health Organization declared that workplaces should be a priority for health promotion [3]. Workplace health promotion entails employers, workers, and communities working together to improve workers’ mental and physical health and overall well-being [4]. Elaborately designed workplace health promotion not only improves workers’ health [2] but also positively affects their productivity [5]. The primary challenge in workplace health promotion involves how to increase worker participation, given that less than 50% of participants typically remain in workplace health promotion programs [6] and the median attrition rate is 28% [7]. These obstacles can be overcome by incorporating health care technologies (eg, electronic health, mobile health [mHealth], wearable devices) into workplace health promotion strategies [8].

Health care technologies can increase workers’ interest, motivation, and participation in workplace health promotion [9,10]. These technologies function as cost-effective health promotion and disease prevention mechanisms by allowing workers to monitor their own health. Workers are at increased risk from stress caused by heavy workloads and unhealthy lifestyles, including lack of exercise and frequent drinking, relative to the general public [11]. In particular, office workers sit for long periods of time in the workplace, which exposes them to an increased risk of developing chronic diseases such as heart disease, cerebrovascular disease, and hypertension [12] that are all associated with high mortality rates as the second, third, and ninth most prevalent causes of death in Korea, respectively [13]. The cost associated with the treatment of cardiovascular disease has reached US $6.9 billion [14], which is higher than the US $4.7 billion spent on cancer, as the most prevalent cause of death, and the burden of disease in Korea is high. Moreover, mortality from cardiovascular disease has steadily increased over the past 10 years [15]. Effective prevention and management are essential because cardiovascular disease not only harms workers’ health but also increases medical expenses [16] and contributes to the social burden caused by decreased corporate productivity [17].

Workplace health promotion using health care technology has been shown to improve participants’ physical activity and eating habits [18,19]. Setting goals, supporting self-monitoring, and providing feedback on changes in physical activity and eating habits can be an effective mechanism for workplace health promotion. Various health care technologies have been studied for efficient workplace health promotion application. Mattila et al [20] conducted a 1-year randomized controlled trial to investigate the activity and usefulness of personal health technologies (web services, mobile apps, and personal monitoring devices) that support workplace health promotion. The authors showed that less than 30% of subjects continued to use mobile apps and web technologies, and that the key requirements for personal health technologies were simplicity, integration with everyday life, and clear feedback. Cook et al [9] conducted a randomized controlled trial for 3 months to evaluate the effectiveness of a web-based workplace health promotion program and found that web-based programs were more effective than a print-based intervention for improving diet and nutrition, but not for improving stress and physical activity. Balk-Møller et al [21] conducted a randomized controlled trial for 38 weeks to investigate the motivations of workers involved in web and app-based workplace health promotion and reported that social functions were more popular than personal functions, and social factors motivated continued use.

Choia et al [22] studied workers’ intention to use health care technology through an investigation of construction workers' acceptance of wearable devices (smart vests and wristbands) for occupational safety and health based on the technology acceptance model (TAM) [23]. They found that perceived usefulness, social influence, and perceived privacy risk were related to the intention to adopt wearable devices. Mohadis et al [24] investigated office workers’ acceptance of mHealth apps designed to increase physical activity based on the unified theory of acceptance and use of technology (UTAUT) [25], and found that performance expectancy and social influence had a significant effect on behavioral intention, but not on effort expectation. Sari et al [26] proposed a UTAUT-based conceptual framework to identify factors that influence worker adoption of and intention to use mHealth technology. Technology acceptance of mHealth apps has also been tested in patients with chronic diseases [27], younger adults [28], and health care professionals [29], but related research on workers in workplaces is still in its infancy [30]. Although many mHealth apps have been developed to date, few of these apps have been developed specifically to improve workplace health promotion [31].

Interest in personal health management is rising as aging and the incidence of chronic disease increase [32]. Moreover, active services focused on prevention and health promotion are needed [33] to record lifestyle factors such as exercise, nutrition, and sleep via various wearable devices [34-36] and to measure blood pressure, blood sugar, and weight via personal health devices [37]. Occupational factors such as the workplace environment should also be considered in managing chronic diseases, either by integrating occupational information into the electronic health record (EHR) [38] or implementing the occupational data for health model [39]. Recently, Health Level Seven (HL7) designed a fast health care interoperability resource (FHIR) profile [40] to represent patients’ occupational elements in personal health records (PHRs). PHRs are electronic tools that allow secure access, management, and sharing of health information [41], which is generally monitored by patients [42]. Individuals can check medical records provided by hospitals, monitor information regarding prescribed medicines and test results, and manage exercise and diet information related to health promotion [43]. Patients can use PHRs to reduce additional medical expenses, and disease management, treatment, and prevention activities can be enhanced as cooperation is improved through communication among medical personnel [44,45].

Employers expect workers to participate in workplace health promotion and enjoy the benefits provided by the organization. In this situation, the concept of PHRs is prominent owing to program technology-based attributes [46]. Employers can provide PHRs that motivate workers’ health care [47]. Employer-sponsored PHRs are driven by commercial goals to reduce productivity loss and health insurance costs by promoting a healthy lifestyle [48]. Despite the interest in and expected effects of PHRs, it is difficult to successfully provide these services [49]. Google Health, released in 2008, suspended the service in 2011 because of poor user participation [50], and Microsoft announced in November 2019 that they would stop providing their PHR HealthVault. However, Apple’s HealthRecord offers large-scale services that could be linked to over 200 medical institutions as of February 2019. These services are offered free of charge, but users’ technology acceptance for PHRs remains low and must be addressed [51]. To implement and operate a successful PHR app service in the workplace, employers, policymakers, developers, and planners must be aware of factors affecting workers’ technology acceptance.

Therefore, the purpose of this study was to analyze the factors that influence acceptance of PHR apps in the workplace and examine differences in perceptions surrounding the main factors. We applied a research model that included Bauer’s perceived risk [52] as an independent variable to the UTAUT [25], which is widely used to explain acceptance of new information technology.

### Theoretical Background and Related Works

#### Unified Theory of Technology Acceptance Use

**Figure 1:**
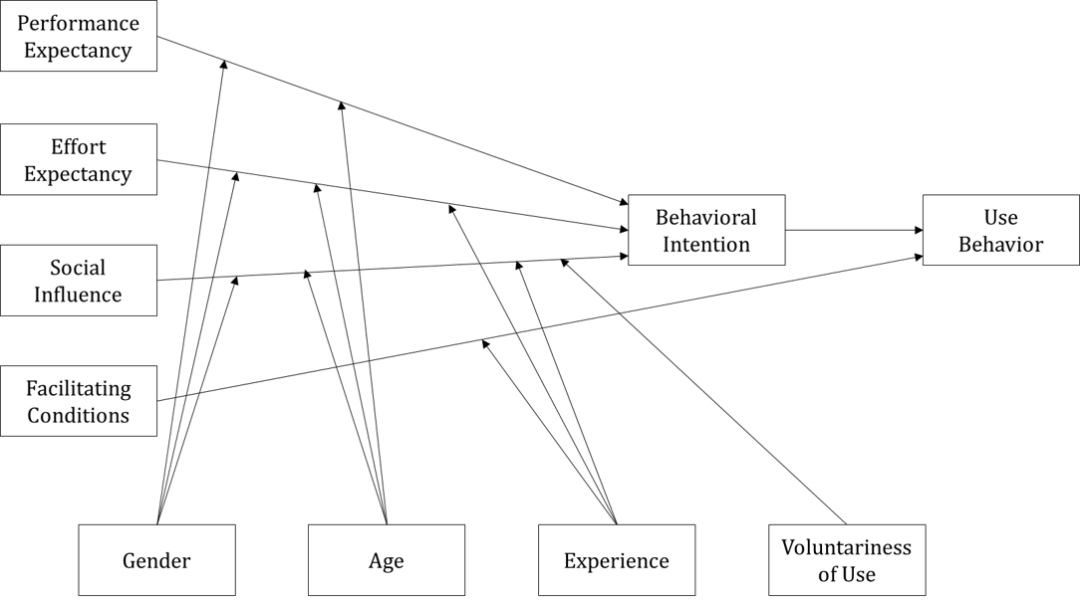
Unified theory of acceptance and use of technology model.

Previous studies examining the acceptance of new information technology adapted the TAM [23] to research technology. However, the TAM does not adequately support the validity of the relationships between exogenous variables, and is therefore suitable for simple technology acceptance studies but has limited ability to analyze interrelationships in complex environments. Venkatesh et al [25] proposed the UTAUT, which assesses users’ technology acceptance from an integrated perspective based on eight representative related theories, including the TAM. The UTAUT model consists of four independent variables (performance expectancy, effort expectancy, social influence, and facilitating conditions) and four moderating variables (gender, age, experience, and voluntariness of use; see Figure 1).

Performance expectancy, effort expectancy, and social influence affect behavioral intention, and facilitating conditions affect use behavior. Performance expectancy is the degree to which system use is perceived to improve work performance; effort expectancy reflects the usability of a system; social influence refers to the degree of awareness that others deemed to be important believe that one should use a new system; and facilitating conditions reflects the degree to which individuals believe that the necessary organization and technical infrastructure are in place to support the use of new systems. These variables are in turn influenced by gender, age, experience, and voluntariness of use when they affect users’ behavioral intention and use behavior.

In a previous study, the UTAUT model explained 70% of behavioral intention and use behavior for an information system, representing a significant improvement in the explanatory power of the model relative to that of existing models, which described 40% of the technology acceptance [25]. Therefore, the UTAUT model can be used to explain users’ technology acceptance of newly developed information technology in medical informatics, which actively converges with other industries. Most previous studies have included electronic medical records [53], PHRs [51], health care devices [54,55], mobile and electronic health services [24,56-59], and telemedicine services [60-63] in the UTAUT model.

#### Perceived Risk

Perceived risk, first introduced by Bauer [52], is the risk subjectively perceived by consumers when performing certain actions such as the uncertainty consumers feel when they cannot predict the outcome of purchase decisions. Short [64] demonstrated that individuals experienced the consequences of this danger; Rayner and Cantor [65] showed the probability of an adverse event occurring and examined subjective assessment of the magnitude of damage incurred by the event. Previous studies [66,67] have examined the effects of perceived risk on users’ acceptance. In addition, previous medical informatics studies [68-70] have examined users’ perceived risk of mHealth technology and electronic medical records.

#### Workers’ Health Center

According to a 2017 analysis of industrial accidents [71] conducted by the Ministry of Employment and Labor, of the 993 deaths due to occupational diseases, 354 involved cardiovascular disease, 215 of which occurred at workplaces with fewer than 50 employees. The risk of cardiovascular disease in workers could be decreased via continuous health management according to the results of medical checkups. However, workers in vulnerable classes often do not benefit from systematic industrial health services because they are rarely offered in small-scale workplaces [72,73]. The workers’ health center was established in Korea in 2011 to meet the rising need for disease prevention and health promotion services in small-scale workplaces.

Workers’ health centers are set up in areas with many small-scale workplaces such as industrial parks, and provide services for preventing occupational disease in workers. There are currently 21 workers’ health centers in operation in Korea, staffed by professional personnel such as occupational and environmental medicine specialists, occupational nurses, industrial hygiene safety engineers, physical therapists, and counseling psychologists, who provide comprehensive occupational health services, including occupational disease prevention, cerebrovascular disease prevention, musculoskeletal disease prevention, workplace environment counseling, job stress prevention, and lifestyle improvement. Workers’ health centers are used by 180,000 workers each year, most of whom are interested in their health care. Workers’ health centers manage a vast amount of worker data through the electronic worker health management system.

#### PHR App

**Figure 2:**
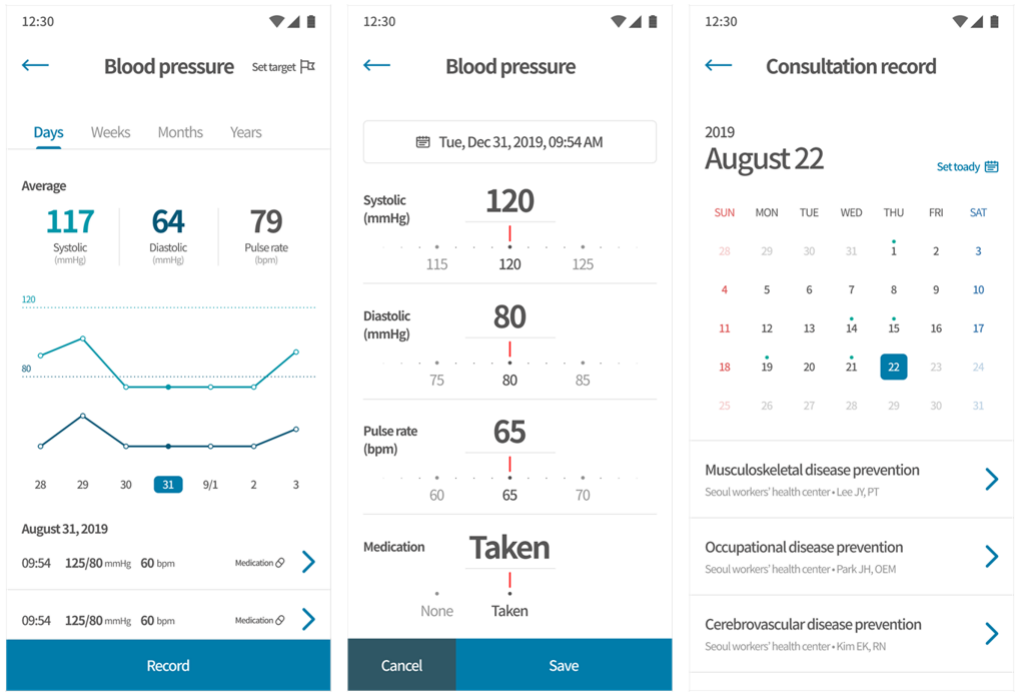
Personal health record app.

Our PHR app (Figure 2) manages a worker’s PHRs (eg, life logs, health information, and medical checkup data) and supports customized health care services and workplace health promotion through links between specific systems and platforms. The worker PHR complies with HL7 FHIR Release 4. The app’s primary functions include data collection, text-based health counseling, consultation reservations, sharing PHRs, and viewing occupational health content. For example, workers can collect their own data stored in the workplace or at the workers’ health center through the PHR app, manage their PHRs, and receive health counseling services from health experts. In addition, by sharing PHRs to specific platforms through self-certification and consent, analysis results (eg, disease prediction, health, and body age) can be confirmed. PHRs based on data collected at workplaces are the basis for continuous health care, regardless of the worker’s external environment (new workplace turnover, local agencies, and hospitals). These data can help decision making for medical personnel by collecting and sharing data among various institutions through our PHR app, which ensures interoperability.

## Methods

### Research Model

The PHR app is an important element of the next generation of health care services and supports personal health promotion by storing and managing important personal medical data in one location. Moreover, using the UTAUT model to analyze and predict users’ technology acceptance of the PHR app is a rational approach. In this study, we set the main variables of effort expectancy, social influence, performance expectancy, and facilitating conditions as factors affecting behavioral intention to use the PHR app based on the UTAUT model. In the model, the dependent variable, use behavior, is affected by behavioral intention and facilitating conditions, and behavioral intention is determined by performance expectancy, effort expectancy, and facilitating conditions. However, as PHRs are currently in the introduction stage in Korea, research on actual users is limited. Therefore, in this study, we assumed that facilitating conditions also affected behavioral intention, and therefore included behavioral intention as a dependent variable without considering use behavior. Gender and age were also assumed to moderate the effects of performance expectancy, effort expectancy, and social influence on behavioral intention.

**Figure 3:**
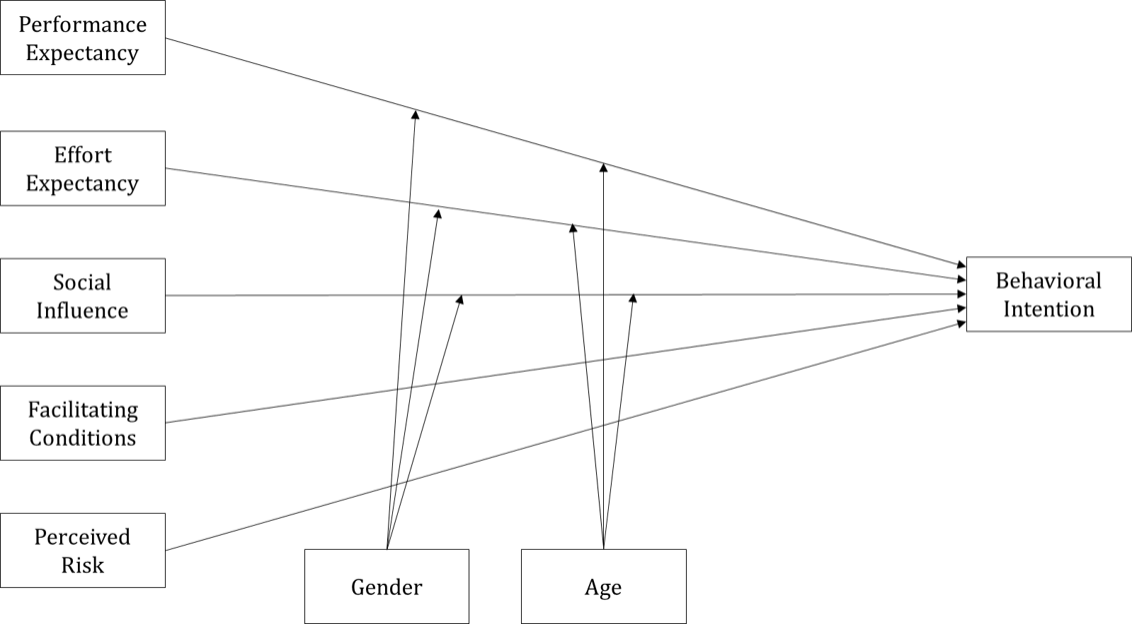
Research model.

The PHR app is accompanied by various risks, which exert direct effects on behavioral intention. For example, medical data collected and utilized by the PHR app contain highly sensitive information, and behavioral intention decreases when there is a high probability of a data breach or fraud. In addition, behavioral intention decreases when users cannot securely manage as much information as they expected, or believe their information will be used for other purposes. These risks should be minimized when introducing health care services based on the PHR. Therefore, this study extended the existing UTAUT model by including perceived risk as a main variable (Figure 3).

### Research Hypotheses

Performance expectancy is defined as “the degree to which an individual believes that using the system will help improve his or her performance” [25]. Performance expectancy is similar to the perceived usefulness of the TAM [23]. In this study, performance expectancy refers to the degree to which users believe that using the PHR app will help them improve their health. The PHR app not only collects and manages the worker’s own data but also improves motivation and participation for workplace health promotion through personalized feedback from health experts [74-77]. Previous studies have demonstrated that performance expectancy and usefulness affect behavioral intention [51,63,78-80]. Therefore, we proposed the following hypothesis, H1: Performance expectancy in the PHR app will exert a positive effect on behavioral intention.

Effort expectancy is defined as “the degree of ease associated with the use of the system” [25], and affects the intent to use and the ease with which users can learn and use the system, similar to the TAM’s perceived ease of use [23] and innovation diffusion theory [81]. In this study, effort expectancy refers to the PHR app’s ease of use; the app should be easy to use, taking workers’ diversity into account (eg, age, type of business). Given that hard-to-use functions have a negative impact on users [82], PHR app functions should make it easy for users to access their data. Previous studies have shown that ease of use affects behavioral intention [51,83,84]. Therefore, we proposed the following hypothesis, H2: Effort expectancy in the PHR app will exert a positive effect on behavioral intention.

Social influence is defined as “the degree to which an individual perceives that important others believe that he or she should use the new system” [25]. In this study, social influence refers to the degree to which users feel that others deemed to be important or work colleagues believe that the user should use the PHR app for enhanced health management. Social influence is associated with organizational culture in the workplace [85], which is known to be an important social characteristic that affects organization, group, and individual behavior [86-89]. Social influence is an important factor in workers’ communications and interactions with their colleagues [90], which has been identified as a suitable factor for technology acceptance in previous studies [55,91-93]. Therefore, we proposed the following hypothesis, H3: Social influence in the PHR app will exert a positive effect on behavioral intention.

Facilitating conditions are defined as “the degree to which an individual believes that an organizational and technical infrastructure exists to support the use of a given system” [25]. In this study, facilitating conditions refers to a user’s belief that an organizational and technical infrastructure exists to support PHR app use. The number of aging individuals is rapidly increasing. Older workers are less capable of acquiring and adopting new technologies such as information and communication technology, and need adequate training and technical support for using such tools. Previous studies have shown that facilitating conditions affect workers’ behavioral intention to use new technology [94-96]. Therefore, we proposed the following hypothesis, H4: Facilitating conditions in the PHR app will exert a positive effect on behavioral intention.

Perceived risk is defined as “the uncertainty due to unforeseen consequences” [52]. In this study, perceived risk refers to the degree to which users are aware of possible loss associated with uncertainty surrounding PHR app use. Since workplaces require insight into the health status of their organizations and workers, personal health data should be strictly protected [97]. The health manager in the workplace handles sensitive medical data such as workers’ medical checkups and data from individuals who have abnormal findings; the PHR app collects and manages these data. Confidence in the system, information privacy, and security concerns affect the sharing behavior of PHRs [98]. Therefore, we propose the following hypothesis, H5: Perceived risk in the PHR app will exert a negative effect on behavioral intention.

In UTAUT, performance expectancy, effort expectancy, and social influence are moderated by gender, age, experience, and the voluntary nature of use [25]. Gender and age are generally perceived as significant factors in attitudes toward information technology [99], which previous studies have treated as moderating variables in technology acceptance [100-102]. Therefore, we proposed the following hypotheses: H6, Effects of performance expectancy on behavioral intention of the PHR app will be moderated by gender and age; H7, Effects of effort expectancy on behavioral intention of the PHR app will be moderated by gender and age; and

H8, Effects of social influence on behavioral intention of the PHR app will be moderated by gender and age.

### Instrument Development

The questionnaire consisted of 42 items. Responses to the 21 items (see Multimedia Appendix 1) measuring the main variables were on a 5-point Likert scale ranging from “strongly disagree” to “strongly agree” (Table 1). In addition, 12 items pertained to participants’ general characteristics and 9 items concerned the experience of and functions required of the app.

**Table 1:** Definition of variables.

Variable	Definition	Number of questions	Reference
Behavioral Intention	The degree of users’ behavioral intention to use the PHR^a^ app	3	[25]
Performance Expectancy	The degree to which users believe that using the PHR app will help them to improve their health	3	[24,25]
Effort Expectancy	Ease of use for the PHR app	4	[24,25]
Social Influence	The degree to which users feel that important others or work colleagues believe that the PHR app should be used for enhanced health management	4	[22,24,25]
Facilitating Conditions	The degree to which users believe that an organizational and technical infrastructure exists to support use of the PHR app	4	[2,25]
Perceived Risk	The degree to which users are aware of possible loss relating to uncertainty surrounding use of the PHR app	3	[52]

^a^PHR: personal health record.

### Data Collection and Analysis

After receiving ethical approval (IRB No. P01-201902-23-014) from the Korea National Institute for Bioethics Policy, we recruited workers and health experts who visited 21 worker health centers with cooperation of the Korea Occupational Safety and Health Agency. The survey included 40 workers and 10 health experts from each worker health center and considered regional distribution.

We used a paper-based questionnaire to verify the proposed research model. The survey was conducted for approximately 7 weeks from February 12 to April 6, 2019. In total, 1050 questionnaires were distributed and 900 were collected. Of the collected questionnaires, we removed 34 that were inappropriate for analysis because they did not meet the purpose of the study or included insincere responses.

Frequency statistics were used to analyze the participants’ general characteristics. Reliability analysis, exploratory factor analysis, and confirmatory factor analysis were used to assess the instrument’s reliability and validity. Correlation analysis was used to examine bivariate associations among the main variables. Multiple linear regression analysis was employed to verify the explanatory power and hypotheses of the research model. Multigroup path analysis was performed to verify the effect of moderating variables, and critical ratios for differences were calculated to verify the statistical significance of the path coefficient for each group. Independent samples *t* tests were applied to analyze differences in perception of the main variables. For frequency analysis and independent samples *t* tests, the participants were divided into workers and health experts, and the remaining analyses were based on all respondents.

We used the factor extraction method, which implements the most commonly used maximum-likelihood estimation, in common factor analysis; for factor rotation, we used the direct Oblimin method in the square rotation to examine correlations between the factors. The determination criterion for the number of factors extracted was an eigenvalue >1. The Kaiser Meyer Olkin index was used to test the suitability of factors for analysis, where values of 0.5-1.0 indicate that the factors are suitable for analysis and those ≤0.5 indicate that the factors are not suitable for analysis. Pearson correlation analysis was performed to examine bivariate correlations before performing multiple linear regression analysis. Correlation analysis was performed using factor score storage values in the factor analysis. In the social sciences, coefficients below ±0.2 are considered low, those between ±0.3 and ±0.7 are considered moderate, and those above ±0.7 are considered high [103]. Analyses were performed with IBM SPSS Statistics ver. 25.0 and AMOS 22 (IBM Corp, Armonk, NY, USA).

## Results

### Participant Characteristics

The study involved 866 participants, including 179 health experts and 687 workers who visited 21 workers’ health centers nationwide. Workers’ general characteristics are summarized in Table 2. The majority of the workers were women. Most participants were older than 50 years, followed by those in their 40s and 30s. The duration of employment in the workplace was most commonly 1-4 years, and 66.1% (454/687) of workers were employed in workplaces with <50 employees. Clerical and service-based businesses were more common than production and technical businesses.

Health experts’ characteristics are shown in Table 3. Similar to the workers, the majority of respondents were women. The most common age group was 30-39 years and the duration of employment in the workplace was most commonly 1-4 years. The most common type of occupation was nursing, followed by physical therapy, industrial hygiene safety engineering, counseling psychology, occupational and environmental medicine, and other.

**Table 2:** Characteristics of the workers (N=687).

Characteristic		n (%)	
**Gender**			
	Male		266 (38.7)
	Female		421 (61.3)
**Age (years)**			
	<20		2 (0.3)
	20-29		124 (18.0)
	30-39		159 (23.1)
	40-49		165 (24.0)
	≥50		237 (34.5)
**Marital status**			
	Single		207 (30.1)
	Married		462 (67.2)
	Widowed		10 (1.5)
	Divorced or separated		8 (1.2)
**Education**			
	Middle school		34 (4.9)
	High school		190 (27.7)
	College (2 years)		100 (14.6)
	College (4 years)		313 (45.6)
	Graduate school		50 (7.3)
**Time in the workplace (years)**			
	<1		111 (16.2)
	1-4		251 (36.5)
	5-9		138 (20.1)
	≥10		187 (27.2)
**Number of employees in the workplace**			
	<5		82 (11.9)
	5-9		105 (15.3)
	10-29		173 (25.2)
	30-49		94 (13.7)
	50-99		53 (7.7)
	≥100		180 (26.2)
**Type of business**			
	Production		67 (9.8)
	Clerical		271 (39.4)
	Service-based		226 (32.9)
	Technical		54 (7.9)
	Other		69 (10.0)

**Table 3:** Characteristics of health experts (N=179).

Characteristic		n (%)
**Gender**		
	Male	45 (25.1)
	Female	134 (74.9)
**Age (years)**		
	20-29	27 (15.1)
	30-39	104 (58.1)
	40-49	35 (19.6)
	≥50	13 (7.3)
**Marital status**		
	Single	75 (41.9)
	Married	103 (57.5)
	Divorced or separated	1 (0.6)
**Education**		
	College (2 years)	28 (15.6)
	College (4 years)	95 (53.1)
	Graduate school	56 (31.3)
**Time in the workplace (years)**		
	<1	30 (16.8)
	1-4	79 (44.1)
	5-9	43 (24.0)
	≥10	27 (15.1)
**Type of occupation**		
	Occupational and environmental medicine	11 (6.2)
	Nursing	75 (41.9)
	Physical therapy	49 (27.4)
	Counseling psychologist	16 (8.9)
	Industrial hygiene safety engineering	23 (12.8)
	Other	5 (2.8)

### Reliability and Validity Analysis

The results of the reliability and exploratory factor analyses are shown in Table 4. Cronbach alpha values greater than .60 and .90 are generally considered acceptable and highly reliable, respectively. Cronbach alpha values for all variables, excluding perceived risk (.69), were within the recommended range (>0.70), and thus the reliability of the main variables was considered acceptable. The analysis was performed without deleting items because none of the items impaired reliability.

The Kaiser Meyer Olkin statistic was 0.90 and the result of the Barlett test was Chi square_210_=14334.09 (*P<*.001); thus, the factor analysis model was considered suitable. In addition, the cumulative variance was 70.63% and the explanatory power of the six factors was high. All factor loading values were above 0.4, which demonstrated the validity of the overall instrument; therefore, the analysis was performed without additional adjustment.

The fit indices for the research model were as follows: Chi square_174_=819.66 (*P<*.001), goodness-of-fit index=0.91, root mean square residual=0.04, root mean square error of approximation=0.07, normed fit index=0.94, relative fit index=0.93, incremental fit index=0.96, comparative fit index=0.95, Tucker-Lewis index=0.95, and adjusted goodness-of-fit-index=0.88. The adjusted goodness-of-fit-index did not meet the criteria, but the overall model fit was satisfactory, and the other indices met the criteria. The results of confirmatory factor analysis (Table 5) showed that the paths to the observed variables were significant (*P<*.001) for all latent variables. The average variance extracted was >0.50 and the construct reliability was >0.70; therefore, convergent validity was demonstrated. In addition, the discriminant validity ensured that the average variance extracted was higher than the square value of the correlation coefficient (Table 6).

**Table 4:** Reliability and exploratory factor analysis.

Variable		Factor 1	Factor 2	Factor 3	Factor 4	Factor 5	Factor 6	Cronbach alpha
**BI^a^ (loading)**								**.95**
	BI1	0.93	0.02	0.01	–0.02	0.04	–0.01	
	BI2	0.88	0.05	0.04	0.01	0.03	–0.02	
	BI3	0.80	0.01	0.06	0.05	0.06	–0.03	
**EE^b^ (loading)**								**.96**
	EE1	0.01	0.96	–0.02	–0.01	–0.03	0.01	
	EE2	–0.004	0.94	0.01	–0.01	0.01	–0.01	
	EE3	–0.02	0.89	0.02	–0.02	0.09	–0.01	
	EE4	0.04	0.83	0.01	0.06	–0.02	–0.02	
**SI^c^ (loading)**								**.92**
	SI1	–0.03	0.04	0.92	–0.01	–0.02	0.03	
	SI2	0.01	–0.03	0.89	–0.02	0.02	–0.03	
	SI3	0.08	–0.04	0.81	–0.01	0.06	–0.07	
	SI4	0.02	0.06	0.76	0.05	–0.03	0.04	
**PE^d^ (loading)**								**.79**
	PE1	0.09	0.01	–0.07	0.80	–0.01	–0.03	
	PE2	–0.14	–0.02	0.05	0.74	0.05	0.02	
	PE3	0.16	0.05	0.02	0.67	–0.03	–0.02	
**FC^e^ (loading)**								**.87**
	FC1	–0.07	0.07	–0.05	.001	0.88	0.02	
	FC2	0.08	–0.04	–0.07	0.01	0.82	0.02	
	FC3	0.08	0.05	0.07	0.01	0.68	–0.04	
	FC4	0.01	0.03	0.17	0.03	0.62	–0.05	
**PR^f^ (loading)**								**.69**
	PR1	0.09	–0.003	0.06	0.003	–0.09	0.84	
	PR2	–0.05	-0.06	–0.02	0.04	–0.03	0.65	
	PR3	–0.05	0.02	–0.05	–0.05	0.08	0.48	
Eigenvalue		8.03	1.97	1.41	1.37	1.11	0.94	
Variance (%)		38.25	9.37	6.74	6.50	5.29	4.49	
Cumulative variance (%)		38.25	47.61	54.35	60.85	66.15	70.63	.87

^a^BI: behavioral intention.

^b^EE: effort expectancy.

^c^SI: social influence.

^d^PE: performance expectancy.

^e^FC: facilitating conditions.

^f^PR: perceived risk.

**Table 5:** Confirmatory factor analysis.

Latent and observed variables		B	beta	SE	Critical ratio	*P* value	AVE^a^	CR^b^
**BI^c^**							**0.90**	**0.96**
	BI1	1.07	.96	0.02	47.39	<.001		
	BI2	1.06	.96	0.02	47.77	<.001		
	BI3	1.00	.89	N/A^d^	N/A	N/A		
**EE^e^**							**0.87**	**0.96**
	EE1	0.90	.86	0.02	41.82	<.001		
	EE2	1.04	.92	0.02	54.17	<.001		
	EE3	1.00	.96	N/A	N/A	N/A		
	EE4	0.99	.93	0.02	56.61	<.001		
**SI^f^**							**0.75**	**0.92**
	SI1	1.01	.90	0.03	38.50	<.001		
	SI2	0.98	.88	0.03	36.89	<.001		
	SI3	1.00	.89	N/A	N/A	N/A		
	SI4	0.92	.78	0.03	29.17	<.001		
**PE^g^**							**0.72**	**0.88**
	PE1	0.89	.77	0.05	19.19	<.001		
	PE2	0.87	.67	0.05	17.68	<.001		
	PE3	1	.82	N/A	N/A	N/A		
**FC^h^**							**0.65**	**0.88**
	FC1	0.99	.77	0.04	24.14	<.001		
	FC2	1.12	.85	0.04	26.80	<.001		
	FC3	1	.80	N/A	N/A	N/A		
	FC4	0.90	.74	0.04	22.76	<.001		
**PR^i^**							**0.50**	**0.75**
	PR1	1.25	.76	0.10	12.30	<.001		
	PR2	1.27	.74	0.10	12.29	<.001		
	PR3	1	.50	N/A	N/A	N/A		

^a^AVE: average variance extracted.

^b^CR: composite reliability.

^c^BI: behavioral intention.

^d^N/A: not applicable.

^e^EE: effort expectancy.

^f^SI: social influence.

^g^PE: performance expectancy.

^h^FC: facilitating conditions.

^i^PR: perceived risk.

**Table 6:** Discriminant validity analysis.

	BI^a^	EE^b^	SI^c^	PE^d^	FC^e^	PR^f^
BI	1					
EE	.29^g^	1				
SI	.39^g^	.21^g^	1			
PE	.15^g^	.10^g^	.08^g^	1		
FC	.23^g^	.45^g^	.10^g^	.06^g^	1	
PR	.25^g^	.18^g^	.09^g^	.09^g^	.14^g^	1
AVE^h^	.90	.87	.75	.72	.65	.50
CR^i^	.96	.96	.92	.88	.88	.75

^a^BI: behavioral intention.

^b^EE: effort expectancy.

^c^SI: social influence.

^d^PE: performance expectancy.

^e^FC: facilitating conditions.

^f^PR: perceived risk.

^g^*P<*.01.

^h^AVE: average variance extracted.

^i^CR: composite reliability.

### Hypothesis Testing

The results of Pearson correlation analysis showed that all dependent variables and the independent variable in the research model were significantly correlated (Table 7). In particular, perceived risk was negatively correlated with all other variables.

We performed multiple linear regression analysis to verify the effects of effort expectancy, social influence, performance expectancy, facilitating conditions, and perceived risk on behavioral intention. The factor analysis results showed that the regression model was statistically significant (*F*=194.96, *P<*.001), as shown in Table 8. The explanatory power of the regression model was 53.1% (*R^2^*=0.53, *adjR^2^*=0.53). Moreover, no issues were observed with respect to the independence of residuals (D-W=2.04) or multicollinearity (variance inflation factor<10).

The regression coefficients showed that effort expectancy, social influence, performance expectancy, and facilitating conditions exerted significant positive effects on behavioral intention, whereas perceived risk exerted a significant negative effect on behavioral intention (Table 8). Therefore, the results supported H1, H2, H3, H4, and H5.

We performed multigroup path analysis to verify the moderating effects of gender and age (Table 9). In the male group, effort expectancy, social influence, and performance expectancy exerted significant positive effects on behavioral intention. In the female group, effort expectancy, social influence, and performance expectancy exerted significant positive effects on behavioral intention. Only the performance expectancy (critical ratio=–3.38, *P<*.001) showed statistically significant differences in the path between males and females.

In the younger group, effort expectancy, social influence, and performance expectancy exerted significant positive effects on behavioral intention. In the older group, effort expectancy, social influence, and performance expectancy exerted significant positive effects on behavioral intention. Only the performance expectancy (critical ratio=1.97) showed statistically significant differences in the path between younger and older respondents. Therefore, the results supported H6, but H7 and H8 were rejected.

The path analysis of workers and health experts showed that effort expectancy, social influence, performance expectancy, and facilitating conditions exerted significant positive effects on behavioral intention, whereas perceived risk exerted a significant negative effect on behavioral intention. Only the perceived risk (critical ratio=–2.24) showed statistically significant differences in the path between workers and health experts.

**Table 7:** Pearson correlation coefficients among dependent variables.

	BI^a^	EE^b^	SI^c^	PE^d^	FC^e^	PR^f^
BI	1.00					
EE	0.51^g^	1.00				
SI	0.61^g^	0.47^g^	1.00			
PE	0.33^g^	0.20^g^	0.28^g^	1.00		
FC	0.42^g^	0.67^g^	0.28^g^	0.21^g^	1.00	
PR	–0.53^g^	–0.42^g^	–0.32^g^	–0.31^g^	–0.34^g^	1.00

^a^BI: behavioral intention.

^b^EE: effort expectancy.

^c^SI: social influence.

^d^PE: performance expectancy.

^e^FC: facilitating conditions.

^f^PR: perceived risk.

^g^*P <* .01.

**Table 8:** Multiple linear regression analysis with behavioral intention as the dependent variable.

Independent variables	B	SE	beta	t_865_	*P* value	VIF^a^
(Constant)	–6.95E-17	0.02		0.00	>.99	
EE^b^	0.08	0.04	.08	2.17	.03	2.24
SI^c^	0.43	0.03	.43	15.73	<.001	1.34
PE^d^	0.07	0.03	.07	2.67	.008	1.17
FC^e^	0.14	0.03	.13	4.30	<.001	1.81
PR^f^	–0.32	0.03	–.29	-10.99	<.001	1.31

^a^VIF: variable inflation factor.

^b^EE: effort expectancy.

^c^SI: social influence.

^d^PE: performance expectancy.

^e^FC: facilitating conditions.

^f^PR: perceived risk.

**Table 9:** Multigroup path analysis.

Path		Male/ Younger (<39 years)/Workers									Female/Older (≥40 years)/Health experts								Critical ratio for difference
			B		beta		SE		*P* value		B		beta		SE		*P* value		
**Gender (Male or Female)**																			
	EE^a^ to BI^b^		0.10		.09		0.07		.02		0.08		.11		0.05		.02		–0.91
	SI^c^ to BI		0.38		.40		0.05		<.001		0.42		.42		0.04		<.001		–0.62
	PE^d^ to BI		0.04		.06		0.07		<.001		0.14		.16		0.06		<.001		–3.38
**Age (Younger or Older)**																			
	EE to BI		0.07		.06		0.07		.01		0.10		.10		0.06		<.001		0.34
	SI to BI		0.39		.39		0.05		<.001		0.42		.40		0.05		<.001		0.38
	PE to BI		0.13		.12		0.08		.04		0.04		.05		0.06		<.001		1.97
**Group (Workers or Health experts)**																			
	EE to BI		0.08		.08		0.05		.007		0.10		.11		0.01		.03		0.14
	SI to BI		0.38		.40		0.03		<.001		0.45		.42		0.08		<.001		0.75
	PE to BI		0.11		.13		0.05		<.001		0.08		.09		0.01		.03		0.14
	FC^e^ to BI		0.13		.12		0.05		<.001		0.15		.12		0.02		<.001		–1.65
	PR^f^ to BI		-0.18		–.20		0.07		<.001		-0.36		–.38		0.03		<.001		–2.24

^a^EE: effort expectancy.

^b^BI: behavioral intention.

^c^SI: social influence.

^d^PE: performance expectancy.

^e^FC: facilitating conditions.

^f^PR: perceived risk.

### Differences in Perception between Workers and Health Experts

The results of the independent samples *t* tests to analyze differences in perception of the main variables between workers and health experts are summarized in Table 10. Workers’ mean scores for the main variables were higher relative to those of health experts for all variables except perceived risk. Moreover, behavioral intention, effort expectancy, social influence, performance expectancy, and perceived risk differed significantly between groups, whereas facilitating conditions did not.

**Table 10:** Perception differences between groups.

Variable	Health experts, mean (SD)	Workers, mean (SD)	t_864_	*P* value
BI^a^	3.44 (0.97)	3.72 (0.82)	–3.58	<.001
EE^b^	3.43 (0.79)	3.70 (0.83)	–3.90	<.001
SI^c^	2.92 (0.92)	3.34 (0.86)	–5.69	<.001
PE^d^	3.90 (0.58)	4.01 (0.60)	–2.20	.03
FC^e^	3.59 (0.73)	3.63 (0.84)	–0.69	.49
PR^f^	2.84 (0.69)	2.56 (0.69)	4.85	<.001

^a^BI: behavioral intention.

^b^EE: effort expectancy.

^c^SI: social influence.

^d^PE: performance expectancy.

^e^FC: facilitating conditions.

^f^PR: perceived risk.

## Discussion

The purpose of this study was to analyze the factors influencing users’ behavioral intention to use a PHR app and identify differences in perception of the main variables to inform the development of personalized health care services for workers. We developed a research model that added perceived risk to the UTAUT, which is a representative theory that explains information technology acceptance, and conducted an empirical study involving health experts and workers who visited 21 workers’ health centers. After receiving ethical approval from the Korea National Institute for Bioethics Policy, 1050 questionnaires were distributed over 7 weeks to 40 workers and 10 health experts from each workers’ health center; 900 completed questionnaires were collected. The number of respondents in the analysis was 866 (687 workers and 179 health experts).

Performance expectancy exerted significant positive effects on behavioral intention to use the PHR app. These results are consistent with those of previous studies [24,51,56-58,62,63]. Lee et al [56] demonstrated that performance expectancy was an important determinant of app use behavioral intention between college students and workers. Liu et al [57] showed that people outside the normal range of body mass index show the closest relation to performance expectancy with a fitness app’s behavioral intention. Mattila et al [18] showed that workers’ requirements for personal health technologies were simplicity, integration with everyday life, and clear feedback. To increase the behavioral intention of workers on the PHR app, it is necessary to both efficiently represent the causal relationship between physiological conditions associated with the collected data, and provide functions that can help workers’ health conditions through immediate feedback from health experts.

Effort expectancy exerted significant positive effects on behavioral intention to use the PHR app. These results are consistent those of previous studies [51,56,57,59,62,79]. Koivumäki et al [59] showed that effort expectancy was an important factor in consumers’ behavioral intention for electronic health services using personal data. Wang et al [79] showed that effort expectancy affects the intention to use a health care app, regardless of whether or not it is used. Ensuring the ease of use of the PHR app was a top priority for developers and planners because health care services are used at various ages and in various groups; therefore, the design and use of the method must be very intuitive. No matter how much the PHR app offers, if the interface is complex, users will stop using it.

Social influence exerted significant positive effects on behavioral intention to use the PHR app. These results are also consistent with previous studies [24,53,57,58,62,63,83,93]. Tan et al [93] showed that social influence significantly affected the intention to use personal digital assistance devices among medical professionals. Homburg et al [104] showed that bosses’ and colleagues’ opinions affect the intention of subordinates to adopt new technologies. Balk-Møller et al [105] showed that social function such as a peer challenge was used for a longer time to improve workplace health promotion than other app functions. Social influence demonstrates an important role between individuals and groups in the social ecology of workers, such as colleagues, employers, and health care professionals. Employers can influence workers’ behavioral intention by providing an organizational culture that facilitates using the PHR app through health management policies that promote a healthy workplace environment. Workers exerted a synergistic effect on health promotion practices if employers and health managers played an active role in workplace health promotion programs [106]. Considering that social influence exerted the strongest effect on behavioral intention to use the PHR app, the app should include functions that enable interaction between colleagues or health experts.

In addition, workers’ behavioral intention increased when the app was linked to workplace health promotion activities or management direction of community institutions such as workers’ health centers. Workers’ health centers have conducted community institution activities such as occupational health care services for workers, on-site consultation services for the workplace, and establishing cooperative systems through networking among various institutions in the community [107]. Moreover, workers’ health centers provide personalized health care services to individual workers through an understanding of workers’ areas and the characteristics of industrial parks and working environments [108]. To continuously develop workers’ health centers, it is necessary to build practices that systematically collect information regarding workers’ medical checkups and harmful factors in the workplace and apply this information to follow-up management of the centers [107]. Under these circumstances, data collected via the PHR app can be used for personalized health care services and follow-up management of workers’ health centers.

Facilitating conditions exerted significant positive effects on behavioral intention to use the PHR app. These results are consistent with previous studies [53,58,63,83]. Stieglitz and Brochmann [109] proposed that facilitating conditions can be divided into material support (eg, incentives) and nonmaterial support (eg, training). It is difficult to retain workers who are not interested in continuous workplace health promotion participation. If the program emphasizes external motivations such as incentives rather than cycles through which personal motivation can be generated, the degree of participation will initially increase but cannot be sustained in the long run. It is important to configure an infrastructure that can support participants. Health managers in the workplace or clinicians in the clinic can run various health care programs using the PHR app and select individuals with risk factors to help them learn about healthy lifestyles. Employers can influence workers’ behavioral intention by establishing usage training, technical support teams, and organizational policies for the PHR app, along with wearable devices. Iron Mountain, a records and data management company, is running LiveWell [110], a worker health care program that uses wearable devices and apps. The program motivated workers to improve their health through policies that provide workers with various tasks and paid cash points are given to those who complete the tasks.

Perceived risk exerted a significant negative effect on behavioral intention to use the PHR app. The results showed that a greater possibility of loss from using the PHR app was associated with lower behavioral intention. This reflected users’ concerns regarding the potential risks of using the PHR app, including potential personal information breaches. These results are consistent with previous studies [66,70,93], in which perceived risk affected the intention to use a particular technology. Choia et al [19] showed that potential risk factors such as workers’ personal health information and personal location collected from wearable devices affect workers’ intention to adopt the technology. Construction managers have also stressed the importance of addressing privacy issues before introducing wearable devices. Dawson et al [47] emphasized that workers are concerned about trust and confidentiality when accepting PHRs. Burkhard et al [48] noted that workers have concerns about completeness and accuracy as well as the privacy and security of PHRs. Health care technologies in the workplace enable data-based human resource management through health risk assessment for each worker. The employer may prevent disease by following a worker’s condition, but the worker may be concerned about the personal disadvantages of employers obtaining and tracking personal health information. Therefore, employers need to consider the workers’ perspective before introducing a PHR app in the workplace. It is important to gain the trust of workers and protect personal information through transparency of goals and procedures. Additionally, employers need to consider that (1) the purpose of the PHR app should be to facilitate a healthy culture within the workplace through workers’ health promotion and disease prevention, rather than to facilitate personnel management; (2) transparency of procedures, including the purpose and utilization, should be maintained and disclosed to PHR app users; and (3) the PHR app should protect privacy with user-centered design and operation.

To protect and maintain workers’ health, Korean workplaces should provide medical checkups for workers at institutions that have been designated by the Ministry of Employment and Labor or under the national health insurance law. The results of medical checkups are confidential and include not only personal information but also family history, lifestyle habits, previous history, and disease status. This information requires higher levels of protection than that required for general personal information. The results are sent directly to individual workers to strengthen personal information protection; however, this is inconvenient for health managers in the performance of their duties, because workers who lose or do not present their medical checkup results during consultations with health managers do not receive accurate consultations [111]. The PHR app can be used to collect and manage these results and share health information with specific medical personnel with consent. Moreover, it is necessary to develop security devices and specify security responsibilities for each step of medical data processing to increase the behavioral intention to use the PHR app.

The association between performance expectancy and behavioral intention of the PHR app was moderated by gender and age, where performance expectancy was higher in female and younger participants. These results are consistent with previous studies [62]. Wang et al [112] showed that women have a higher preference for health care apps than men. Guo et al [102] argued that younger individuals showed a positive attitude toward the use of new technology, whereas older individuals were often slower to acquire technology. Adas [113] reported that men were more interested in technology than women, but Fitzgerald et al [114] argued that women are more interested in health status and are therefore more likely to seek medical advice and preventive care than men. The present study showed that gender and age did not moderate the effects of effort expectancy, social influence, or behavioral intention. These results are consistent with previous studies [62,99]. Gender and age are important factors in the health care environment, but there is no strong evidence identifying their specific roles [99]. Therefore, future research should focus on moderating variables such as gender, age, educational background, and app use experience.

In addition, behavioral intention, effort expectancy, social influence, performance expectancy, and perceived risk differed between workers and health experts; workers’ mean scores for the main variables were higher than those of health experts. Facilitating conditions scores did not differ between the two groups. Previous studies have identified differences in patients’ and providers’ perceptions, attitudes, and preferences regarding health care technologies [115], including PHRs [116]. The present study identified differences in perspective between consumers and providers regarding PHRs. PHRs are similar to EHRs in that they collect and manage individual health-related information in one place, but can be distinguished from EHRs in that individuals have ownership or control of the information. That is, providers can obtain information from PHRs only when authorized through access controls set by the consumer. According to related studies, patients with a strong need for clinical services who had chronic diseases with complications were highly likely to use PHRs [117-119], and most empirical studies have shown that patients were highly satisfied with their PHRs [119-121]. In contrast, some studies have shown that doctors were less likely to expect benefits from their patients’ use of PHRs and were concerned about the impact of PHR use on their workloads [122,123]. However, the workload burden resulting from PHR use was found to be lower than expected, and medical personnel were generally satisfied with PHRs [120,121,124]. Since PHRs are currently in the introduction stage in Korea, most health care professionals, including health experts at workers’ health centers, have no practical PHR experience. As in previous studies, concerns about increased workload, record accuracy, and the negative impact on patients from information disclosure are judged to have affected health experts’ relatively low confidence in PHRs. PHR apps could help health care providers make decisions and provide information based on consumers’ health records; however, they should only be implemented after conducting sufficient research examining necessary information collection and functions to ensure a balance between providers’ and consumers’ needs.

This study is the first to examine the factors influencing behavioral intention to use a PHR app in the field of occupational safety and health in Korea. Most studies have focused on the intention of patients, the elderly, and providers to use EHRs, health care devices, and telemedicine services; however, few have analyzed the intention to use PHR apps for workplace health promotion. This study is meaningful in that it reports on workers’ and health experts’ acceptance of interconnected PHR apps to improve workplace health promotion, but it also has some limitations. For example, the study included workers who visited workers’ health centers; therefore, data were collected mainly from workplaces with fewer than 50 employees. In addition, the health experts offering health care services at workers’ health centers are limited to workers in workplaces with fewer than 50 employees, and these small-scale employers are not obliged to appoint health managers. Consequently, the actual work performed by health managers may differ from that of workers’ health center health experts. To derive more generalizable research results, future research should include workers and health managers from different sized workplaces. In addition, the study included only basic participant characteristics such as gender and age. Future studies should examine behavioral intention to use PHR apps according to users’ health status, disease, experience, and working environments. Moreover, PHRs are currently in the introduction stage in Korea, and there has been minimal scholarly debate regarding the use of workers’ PHRs. The current results could change according to the purpose and function of PHRs. Therefore, future research should examine the functions and application range of PHR apps for workers and health managers. Further, this study focused on acceptance of the PHR app for workplace health promotion through a research model that added perceived risk to the UTAUT, but it is also necessary to analyze behavioral changes in health promotion facilitated by PHR app use. Therefore, future research will analyze changes in workers’ health promotion behavior associated with workplace PHR app services by applying the health belief model [125], a representative theory that explains changes in health behavior. Future study will also analyze workers’ usage logs collected through the service operation as well as participants’ lifestyle changes and risk factor changes associated with metabolic syndrome and service satisfaction.

## Appendix

Multimedia Appendix 1 Overview of the instrument.

## Abbreviations

EHR: electronic health record
FHIR: fast healthcare interoperability resource
HL7: Health Level Seven
mHealth: mobile health
PHR: personal health record
TAM: technology acceptance model
UTAUT: unified theory of acceptance and use of technology
