# Supplementary material for: Factors Influencing Acceptance of Personal Health Record Apps for Workplace Health Promotion: Cross-Sectional Questionnaire Study
Source: JMIR Mhealth Uhealth. 2020 Jun 4;8(6):e16723. doi: 10.2196/16723 (PMC7303838; doi:10.2196/16723)
Supplement: Multimedia Appendix 1 [file mhealth_v8i6e16723_app1.docx]

## Multimedia Appendix 1

Overview of instrument.

| Behavioral intention | - I intend to use or continue to use personal health record apps. - I plan to use personal health record apps frequently. - I have a high intention to use personal health record apps. |
| --- | --- |
| Performance expectancy | - Personal health record apps help me to monitor my health status. - Personal health record apps educate me in how to deal with my diseases. - Personal health record apps are useful in managing my health status. |
| Effort expectancy | - My interaction with personal health record apps is clear and understandable. - It would be easy for me to become skillful at using the personal health record apps. - I find personal health record apps easy to use. - Learning how to use personal health record apps is easy for me. |
| Social influence | - People who influence my behavior (e.g., work colleagues, employer) think I should use personal health record apps. - People who are important to me (e.g., family members) think I should use personal health record apps. - People whose opinions that I value (e.g., health managers, physicians) think I should use personal health record apps. - My workplace has supported the use of personal health record apps. |
| Facilitating conditions | - My workplace has the policy (e.g., incentive) to support using personal health record apps. - My workplace has the resources (e.g., health promotion program) to support using personal health record apps. - I have the knowledge necessary to use personal health record apps. - I can get help from others when I have difficulties using personal health record apps. |
| Perceived risk | - I would not feel secure managing personal information on personal health record apps. - I think my personal information will be used for other purposes if I use personal health record apps. - I face the risk of personal information leakage if I use personal health record apps. |
| 1=strongly disagree, 2=disagree, 3=neither agree nor disagree, 4=agree, 5=strongly agree | |
